# A Novel Technique of Renorrhaphy in Difficult Partial Nephrectomies by Single-Layered Parenchymal Imbrication

**DOI:** 10.7759/cureus.42702

**Published:** 2023-07-30

**Authors:** Deepak Raghavan, Deerush Kannan, Praveen G Sekaran, Mathisekaran Thangarasu, Sanjay Prakash J, Rajesh Paul, Pratik Taur

**Affiliations:** 1 Urology, Apollo Hospitals, Chennai, IND

**Keywords:** parenchymal imbrication, single layer, single layer parenchymal imbrication, nephrectomy, robotic partial nephrectomy, open partial nephrectomy, new surgical technique

## Abstract

Background

In partial nephrectomies, achieving the trifecta outcome of negative tumor margins, no surgical complications, and minimal decline in renal function depends on various factors, with the complexity of the tumor described by the nephrometry score being chief among them. These factors often motivate surgeons toward a minimally invasive route even if the preferred route is an open approach. We describe an innovative renorrhaphy technique that overcomes the commonly encountered difficulty in reconstructing the renal parenchyma after resecting a complex tumor with a single-layered parenchymal imbrication (SLPI) technique.

Methodology

We conducted a retrospective review of case records of the patients who had undergone partial nephrectomies in our center from March 2017 to March 2021. The patients who underwent the SLPI technique were chosen, and data were extracted. Data collected included patients' preoperative imaging findings; intraoperative parameters such as ischemia time, blood loss, and number of renal arteries; and postoperative factors such as margin positivity rate, urine leak, secondary bleeding, follow-up imaging, and recurrence rates.

Results

A total of 28 patients were included in our study. The estimated blood loss was 234 mL (standard deviation [SD] = 55 mL), warm ischemia time was 31 minutes (SD 4 minutes), a hospital stay of 3 days (SD 2 days), two minor complications, two intraoperative complications, and one margin positivity. There were no major complications or recurrences.

Conclusions

The novel technique of SLPI renorrhaphy can help deal with complex renal masses and is an easily reproducible technique both in open and minimally invasive approaches.

## Introduction

Partial nephrectomy is the recommended treatment for T1 renal masses and can be successful for certain cases of T2 tumors [1,2]. Achieving the trifecta outcome in partial nephrectomies, which involves negative tumor margins, nil surgical complications, and minimal decline in renal function, depends on factors such as parenchymal loss and ischemia time [3]. The nephrometry score that comments on the complexity of the tumor is a crucial factor affecting the trifecta outcome [4]. While surgeons typically prefer an open approach for complex tumors, a minimally invasive approach can be utilized if the need for radical conversion is low and a successful trifecta outcome can be achieved [5]. Our innovative renorrhaphy technique overcomes the commonly encountered difficulty in reconstructing the renal parenchyma after resecting a complex tumor. This technique has proven to be lifesaving in cases where approximating the renal parenchyma after the inner layer renorrhaphy is often challenging.

In this study, we present a detailed description of our reconstruction steps using the single-layered parenchymal imbrication (SLPI) technique, discuss its ideal applications, and report the follow-up outcomes based on our initial experience with 28 cases that were performed by open and minimally invasive approaches.

## Materials and methods

This study was performed at our center in the Department of Urology from March 2017 to March 2021. The study adhered to the guidelines outlined in the Declaration of Helsinki and received approval from the Institutional Ethics Committee (IRB number: AMH-C-S-013/02-23). The case records of 28 patients in whom the SLPI technique was used were selected for the study. The decision to use the SLPI technique for the second-layer renorrhaphy was made by the operating surgeon, taking into consideration the preoperative imaging findings and the defect in the tumor bed after resection. Written consent was obtained from all patients as a part of the preoperative process for any surgery.

All procedures were performed by the same surgeon. We extracted data, including patients' preoperative imaging findings; intraoperative parameters such as ischemia time, blood loss, and number of renal arteries; and postoperative factors such as margin positivity rate, urine leak, secondary bleeding, follow-up imaging, and recurrence rates. The demographic data, including age, gender, side of the tumor, mean preoperative serum creatinine, and mean preoperative estimated glomerular filtration rate (GFR), were also recorded. 

Steps of renorrhaphy

**Figure 1:**
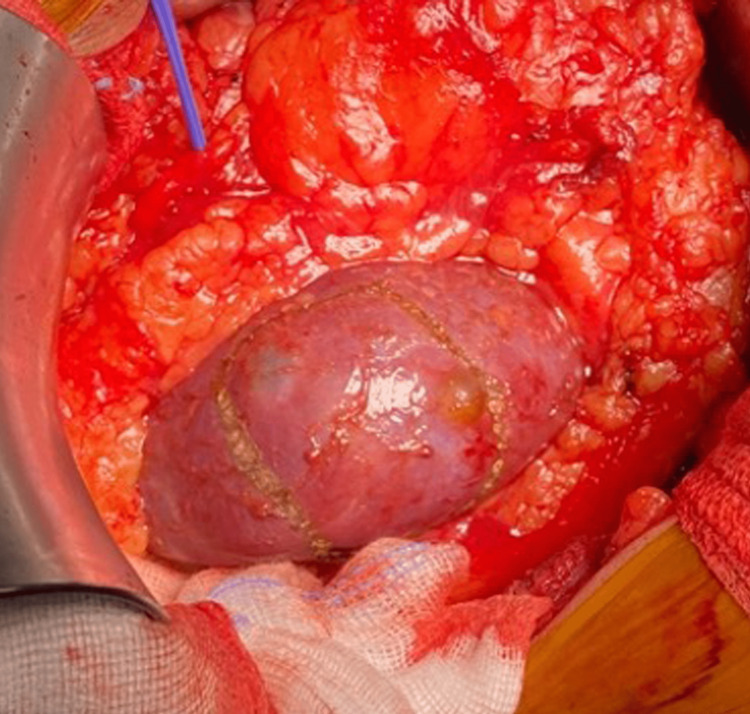
Margins for excision marked using electrocautery.

**Figure 2:**
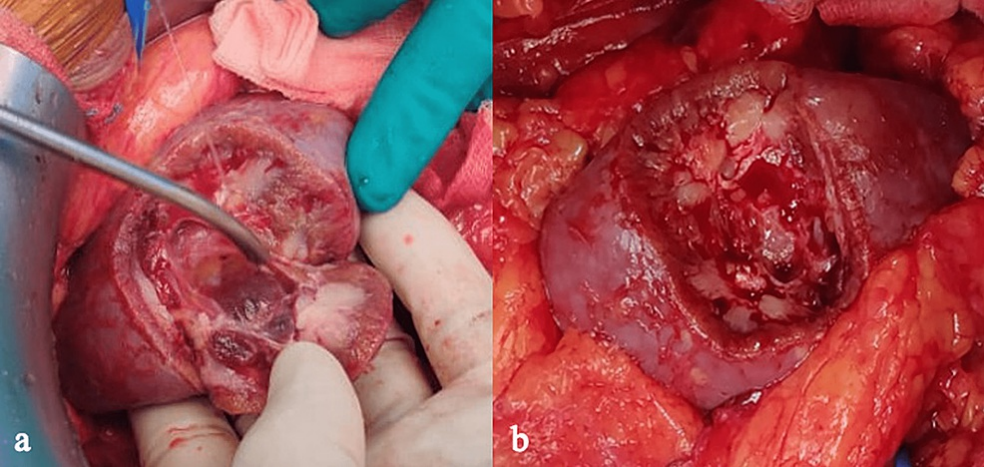
(a) Tumor resection; (b) tumor bed post-resection.

Following the completion of hilar dissection and looping of the renal vessels, Gerota's fascia was opened and the tumor was visually identified [6]. Intraoperative ultrasound was used to confirm the extent of the tumor. The margins of the tumor were marked using monopolar cautery (Figure 1). Subsequently, the renal artery was clamped (the renal vein was not clamped to ensure retrograde perfusion), and the tumor was resected with sufficient margins in the enucleative resection plane, with the utmost care taken to preserve the surrounding healthy parenchyma while ensuring adequate tumor margins (Figure 2) [7]. The steps of this technique in both open and robotic approaches were identical.

**Figure 3:**
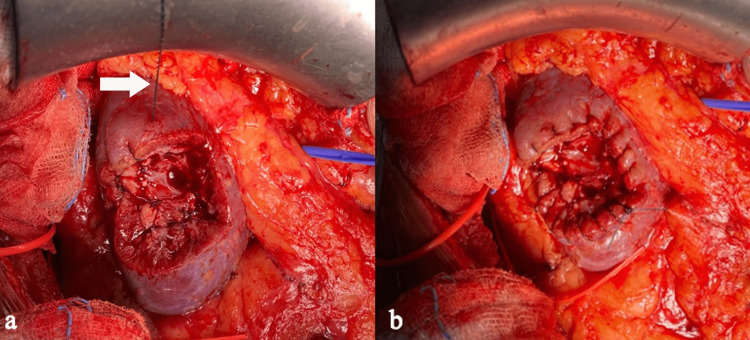
(a) Our technique of SLPI (white arrow showing the suture); (b) the final appearance before re-approximation of Gerota’s fascia. SLPI, single-layered parenchymal imbrication

Each vessel at the tumor base was individually sutured using figure-of-eight sutures with 4-0 Vicryl (Ethicon, Raritan, NJ, USA). In cases where the defect was large that hindered adequate closure of the parenchyma, the SLPI technique renorrhaphy was performed. The corticomedullary area that was left raw after the tumor resection was sutured with 2-0 V-Loc (Medtronic, Minneapolis, MN, USA) sutures, with the needle entering from the cortex into the medulla and reentry at a point 5 mm away from the initial cortical entry again running from the cortex to medulla (Figure 3). In this manner, suturing was conducted to cover the entire raw area of the exposed cortex and medulla, thereby preserving the tumor bed. The suture end was brought out from the normal renal parenchyma on the opposite side of the initial entry and secured with a Hem-o-Lock clip. At this point, the clamp was released, and any minor bleeding was individually suture-ligated.

We introduced a 20-minute waiting period after renorrhaphy, during which the patient's blood pressure was maintained at their baseline by the anesthetist. Afterward, the Gerota's fascia was reapproximated using an absorbable suture, and the closure process began.

## Results

Our study included 28 patients who underwent renorrhaphy with the SLPI technique. The demographic data are given in Table 1. Intraoperative complications were noted in two patients, which involved bleeding post-clamp release. These incidents were managed by suture ligation of the individual bleeding vessels. Minor complications, such as increased serosanguinous drain output, were noted in two patients. However, they were successfully managed with delayed ambulation, and the issues were settled by the second postoperative day without any intervention. No major complications, such as urine leak or bleeding requiring transfusion, were observed. One patient had a positive tumor margin on the final histopathology report (Table 2).

**Table 1:** Demographics and preoperative findings. eGFR, estimated glomerular filtration rate

Demographics	Preoperative findings
Patient total (n)	28
Mean age (years)	50.2
Sex (n)	
Male	17
Female	11
Mean preoperative creatinine (mg/dL)	0.9
Mean preoperative eGFR (mL/minute per body surface area)	80.2
Mean RENAL nephrometry score	10.5

**Table 2:** Intra- and postoperative parameters. GFR, glomerular filtration rate

Parameter	Postoperative parameters (*n* = 28)
Estimated blood loss (mL)	234
Warm ischemia time (minutes)	31
Minor complications	2
Major complications	0
Blood transfusion	0
Intraoperative complications	2
Hospital stay (days)	3
Positive surgical margin	1
Mean postoperative GFR at three months follow-up (mL/minute per body surface area)	68.2

## Discussion

Our study focuses on describing a novel technique for renorrhaphy in complex nephrectomies; therefore, we specifically included patients with complex renal masses in our analysis. In a meta-analysis conducted by Sharma et al., the authors specifically looked at patients with a RENAL score higher than 10 and measured various parameters such as surgery duration, warm ischemia time, estimated blood loss, and length of hospital stay [8]. The results were similar to our study. Among our patients, we encountered no major complications, and only two minor complications were observed, as described by Dindo et al. [9]. The specific technique of renorrhaphy was not mentioned in the meta-analysis as it reviewed multiple studies.

In our study, we used the described technique of SLPI renorrhaphy and found similar results to the meta-analysis in terms of surgery duration and minor complications. However, our study showed lower rates of margin positivity and major complications. We did observe a marginal increase in ischemia time compared to the meta-analysis. However, our approach involved selectively clamping only the renal artery. In some cases, we performed selective clamping of arteries based on tumor location and vascularity. To ensure retrograde perfusion, the renal vein was not clamped in any patient. We did not observe a significant decline in renal function during the one-year follow-up, suggesting that our clamping principle could be one of the factors that help prevent the decline of renal function. Importantly, we did not encounter any cases of delayed bleeding or pseudoaneurysm formation in our study.

In their study, Chavali et al. reported a pseudoaneurysm rate of 1.4% in a study involving 1,417 patients [10]. The presence or absence of pseudoaneurysm was not associated with differences in age, baseline renal function, or RENAL nephrometry score in their study. However, patients who developed pseudoaneurysms had longer operative times (225.6 minutes versus 193 minutes) and cold ischemia times (48 minutes versus 29 minutes) [8]. While our ischemia time was similar to this study, our technique of selectively suturing individual vessels could potentially reduce the likelihood of pseudoaneurysm formation as noted in our study.

To ensure that there were no postoperative bleeds, we implemented a 20-minute waiting period after renorrhaphy while maintaining the patient's blood pressure at their baseline. This precautionary measure could add to reducing the chances of further postoperative bleeding. Furthermore, we did not encounter any cases of urinary leaks, which may be attributed to our suturing of the calyces.

In summary, our study with its specific renorrhaphy technique yielded comparable results to the meta-analysis in terms of surgery duration and minor complications. We observed lower rates of margin positivity and late complications, although there was a marginal increase in ischemia time. Our approach to individual suturing of vessels may contribute to the prevention of pseudoaneurysm formation. However, as a retrospective study, there could be potential selection bias, and the relatively small sample size, resulting from the technique being employed only in certain cases of partial nephrectomies, are notable limitations of the study.

## Conclusions

The novel technique of SLPI renorrhaphy can help in managing complex renal masses, particularly in instances where traditional renorrhaphy methods may present significant challenges. It’s an easily reproducible technique both in open and minimally invasive approaches. The initial results are promising in terms of achieving the trifecta. Furthermore, conducting large-scale studies and comparing it with commonly performed renorrhaphy techniques can help further validate and apply the SLPI technique.
